# The Effect of the Built Environment on Older Men′s and Women′s Leisure-Time Physical Activity in the Mid-Scale City of Jinhua, China

**DOI:** 10.3390/ijerph18031039

**Published:** 2021-01-25

**Authors:** Jiabin Yu, Chen Yang, Shen Zhang, Diankai Zhai, Aiwen Wang, Jianshe Li

**Affiliations:** 1 Faculty of Sport Science, Research Academy of Grand Health, Ningbo University, Ningbo 315211, China; nbuzhangshen@outlook.com (S.Z.); zdk5780245@outlook.com (D.Z.); wangaiwen@nbu.edu.cn (A.W.); lijianshe@nbu.edu.cn (J.L.); 2 Department of Kinesiology and Physical Education, McGill University, Montreal, QC H2W 1S4, Canada; chen.yang4@mail.mcgill.ca

**Keywords:** built environment elements, walkability, aging over 60 years old, gender difference, recreational physical activity

## Abstract

Physical activity has been suggested to be beneficial in preventing disease and improving body function in older people. Older people’s leisure-time physical activity (LTPA) is affected by various factors, especially environmental factors. However, the differences in the association between older people’s LTPA and the built environment in different sex groups remain unclear. Perceived built environment scores and older people’s LTPA were collected for 240 older people in Jinhua using the Neighborhood Environment Walkability Scale and International Physical Activity Questionnaire, respectively. A linear regression method was used to analyze the associations between older people’s LTPA and the built environment in men, women, and all participants. The results showed that land use mix diversity was associated with LTPA in older people for both sexes. In men, LTPA was also associated with access to services. However, in women, LTPA was associated with residential density, street connectivity, and crime safety. The relationship varied when demographic variables were incorporated into the regression analysis. Those results indicated that a shorter perceived distance from home to destination would motivate older people to engage more in LTPA. Older people’s LTPA was affected by various built environment factors according to different sex groups. Women’s LTPA was generally more sensitive to the built environment. More studies are needed to confirm the association between LTPA in older people and the built environment in men and women in mid- or small-sized Chinese cities in the future.

## 1. Introduction

Population aging is becoming a global social problem. According to the 2019 Revision of World Population Prospects, 16% of people in the world will be over 65 years old, and the number of people at or over 80 is expected to triple by 2050 [1]. The population aging problem is even more serious in China. By the end of 2040, the percentage of older people over 60 years old will be 24% [2]. The prevalence of diseases, chronic health issues and functional losses greatly increase with aging [3]. Physical activity has been suggested to play an important role in preventing diseases and improving body function by previous studies [4,5,6,7,8,9,10]. Also, as the World Health Organization (WHO) has stated, the benefits of physical activity include lower risks of coronary heart disease, hypertension, stroke, diabetes, colon and breast cancer, enhanced cardiorespiratory and muscular fitness, and so on [11]. Additionally, older people obtain more health benefits from leisure-time physical activity (LTPA) due to greater energy consumption [12]. 

LTPA refers to body movements that involve energy expenditure caused by skeletal muscle contraction in leisure time [13], and it is influenced by various factors [14]. Socio-ecological models indicate that physical activity is affected by internal factors (e.g., lack of interest, fear of falling), social support (by peers or family) and environmental factors (e.g., weather and access to infrastructure) [14]. Meanwhile, as the person-environment-occupation model suggests, environment should be seen from a broad perspective including the cultural, socio-economic, institutional, physical and social considerations of the environment from the perspectives of the person, household, neighborhood or community [15]. For older people, fears about safety, health problems, and lack of guidance from healthcare professionals have been suggested as barriers to physical activity [16,17]. In a systematic review, Baert et al. concluded that barriers to physical activity at the intrapersonal level are quite similar for younger and older adults except for fear, which seems to be a more specific barrier for older people [18]. Motivators for older people’s participation in physical activity include health benefits, lifelong activity and social support [19]. Because older people are often single or widowed (socially isolated), exercise programs involving social interaction may be especially effective in engaging them in physical activity [20].

There is plenty of scientific evidence showing that the built environment is related to the physical activity of residents [21,22,23,24,25,26]. However, few studies have focused on whether this association differs by gender. In a review study, Smith et al. found that older females used parks and greenspaces at a lower frequency than older males did, and parks and greenspaces only promoted older people’s LTPA in males [27]. Sun et al. found that men’s LTPA was associated with street connectivity, walking/cycling facilities and aesthetics, but no significant association was found in females in China [28]. Additionally, Jefferis et al. found a positive association between access to social and leisure actives for males and a nil association for females [29]. While there are a vast number of studies aimed at diverse ages and countries, more studies are needed to investigate the gender differences in the association between built environment and LTPA. A recent report published by the World Bank in 2020 suggested that with women occupying just 10% of the highest-ranking jobs in the world’s leading architectural firms, cities are always built for men and neglect the needs of women. However, people have different needs and routines when it comes to access to the city. Six elements of the built environment combined with gender inequity were mentioned in the World Bank report including access, mobility, safety and freedom from violence, health and hygiene, climate resilience and security of tenure [30]. On the other hand, the model used in the regression analysis would also affect the associations. In Wu et al.’s study, the geographical location of the destination was significantly related with older people’s LTPA in Nanjing, but no significant relationship was found after adjustment by demographics variables [31]. 

The relative Chinese studies have usually been conducted in super large-scale cities like Shanghai [32], Shenzhen [33] and Hongkong [34,35] and large-scale cities like Hangzhou [36], Nanjing [31], and Xi’ an [28]. All the above cities are in the top 20 cities in terms of economic development and population size. Only a few studies have been aimed at cities of middle scale [37]. The participants’ demographic characteristics and social-economic status are definitely different between cities of diverse scale. This would affect the relationship between physical activity and the built environment as well. Yu et al. found that the relationships were different between a first-tier city, Hangzhou and a second-tier city, Wenzhou. All factors varied between cities except for crime safety and walking/cycling facilities [37]. To our knowledge, only one study has targeted middle-size cities in China [37]. Hence, more studies are needed to provide information on mid- or even small-scale cities in China. 

In summary, the objective of this study was to investigate the association between older people’s LTPA and the built environment in a mid-scale city, Jinhua in China. We especially focused on the differences in the associations in different sex groups. Additionally, we also wanted to examine whether the linear regression model results would differ when demographic variables were added as covariables. The findings of this study could help to quantify the influence of the built environment on older people’s LTPA in a more accurate and reliable way and would provide support for decision-making in the urban construction process. 

## 2. Materials and Methods 

### 2.1. Sample and Study Design

This study was conducted in the city of Jinhua, which is located in Zhejiang province in the southeast coastal area of China. It is the fourth largest city in Zhejiang province and ranked 49th in 2019 in terms of economic development in China. The official resident population of Jinhua was 5,624,000 in 2019. Both economic development and the resident population in Jinhua are smaller than those cities used in previous Chinese studies [28,31,32,33,34,35,36,37]. Jinhua is a national-level historic and cultural city and a famous tourism city. The largest Chinese film and television studio, the Hengdian World Studio is located here and is open for tourism. Jinhua is also one of the top ten habitable cities in China. Moreover, it has been awarded with a national title as a sanitary city and it has been set as an example of a law-based governed city. For public transportation, the bus is the main traffic mode for residents in Jinhua and two subway lines are under construction. 

The study was conducted in Wucheng district in Jinhua, which is the downtown area of Jinhua according to its geographical location. Older participants from nine diverse communities were recruited with the assistance of community resident committees. A cross-sectional survey of random samples of older people was conducted. The inclusion criteria for participants in this study were (a) older people over the age of 60 years, (b) residents of the selected communities and have lived there for at least 6 months, and (c) have normal communication abilities and without cognitive impairment. All the questionaries were collected from July to December in 2019, and collected in a one-to-one interview by three team members to ensure the quality. All of them were trained to get familiar with the interview procedure and to understand all the interview questions precisely before they carried out the interview. 

### 2.2. Measures

Demographic data of participants were collected using an individual characteristic questionnaire including gender, age, education level, income situation, travel mode choice and motion sickness. LTPA data for the past seven days were obtained by using the International Physical Activity Questionnaire-Short version (IPAQ-S) [38]. Metabolic equivalent scores (MET) were computed according to the IPAQ scoring procedure. Perceived scores of built environments were collected using the Chinese version of the Neighborhood Environment Walkability Scale-Abbreviated (NEWS-A). The reliability and validity of NEW-A has been shown by a research team from Hongkong [39] and has been widely used in previous studies [28,32,34,35,36,37].

Eight built environment elements were evaluated using the NEW-A. A detailed description of this questionnaire can be found in our previous paper [37]. In brief, the diversity of the land use mix evaluated the distances from home to 20 destinations. Thirty-one questions were included in respect to the other seven built environment elements in total. The average score represents the older person’s perceived score in respect to each built environment element. 

### 2.3. Statistical Analysis

Descriptive statistical analyses were conducted to describe the demographic characteristics in different sex groups. The Chi-square test was conducted to locate the differences in demographic characteristics between sex groups. The differences in older people’s LTPA and perceived scores of built environments in sex groups were examined using the independent *t*-test. A multivariate linear regression method was used to analyze the relationship between older people’s LTPA and built environment components in males and females separately, and then in all participants together. We also analyzed the association between older peoples LTPA and built environment with demographic variables as covariables. Statistical significance was set at *p* < 0.05. SPSS 19.0 software was used to conduct all the analyses (SPSS Inc., Chicago, IL, USA).

## 3. Results

Table 1 shows the demographic characteristics of the participants in different sex groups. Significant differences were found in education level (X_2_ = 21.17, *p* < 0.001), income (X_2_ = 53.41, *p* < 0.001), and travel mode choice (X_2_ = 11.07, *p* = 0.004) between men and women. The education level of most participants was primary or below in both male and female groups. However, the percentage of lower secondary in men was much larger than that in women (42.4% vs. 18.5%), which means that the education level of men was higher than that of women. This result is in line with a previous study in which Zhang found that the education level of older men was higher than that of older women on a national level [40]. With regard to income situation, the income of men was significantly higher than that of women, 33.3% of female participants’ income was 1501–2500 RMB with only 3% of male participants at this income level, and 30.3% of male participants’ income was 3501–4500 RMB, with only 18.5% of female participants at this income level. A previous study also found that the income of older men was higher than that of older women on the national level [41]. The main travel mode was car or bus in both sex groups, but more women chose bicycle (22.2% vs. 12.1%) or walking (14.8% vs. 6.1%) as their travel mode than men. No significant differences were found in age and motion sickness between different sex groups.

Table 2 shows the results of a comparison of older people’s LTPA and perceived built environment scores in different sex groups. Men’s LTPA level was relatively lower than women’s LTPA level (2789.27 vs. 3137.22), without significant difference. Regarding perceived built environment scores, significant differences were found in residential density, access to services, and land use mix diversity. Except for access to services, both the perceived scores for residential density and land use mix diversity in the female group were higher than those in the male group. 

Table 3 shows the association between the built environment and older people’s LTPA in different sex groups. Our results showed that older women’s LTPA was more easily affected by the built environment, and older people’s LTPA was affected by various built elements in different sex groups. In the female group, four built environment elements were significantly associated with older people’s LTPA, including residential density, street connectivity, crime safety, and land use mix diversity. Except for land use mix diversity, all the other three built environment elements were positively associated with older females’ LTPA. Land use mix diversity was negatively associated with older females’ LTPA, which means that a closer distance from home to destination would result in a higher level LTPA in older people. However, in the male group, only two built environment elements were significantly associated with older people’s LTPA. Access to services was positively related with older males’ LTPA. Land use mix diversity was negatively related with older males’ LTPA, which is the same as in the female group. 

Table 4 shows the association between the built environment and older people’s LTPA with the demographic variables included in the linear regression model. For men, access to services and land use mix diversity were still associated with older people’s LTPA. Additionally, crime safety became a positive influence on older people’s LTPA. No significant associations between demographic variables and older males’ LTPA were found. The association changed a little more in the female group compared with the male group. For women, residential density and land use mix diversity were still significantly associated with older people’s LTPA, but the association between street connectivity, crime safety and LTPA became insignificant. Moreover, aesthetics was positively related with older women’s LTPA as a new influence. A significantly negative association was also found for income. 

Table 5 shows the association between the built environment and older people’s LTPA in all participants, without considering sex differences. The results showed that four built environment elements were significantly associated with older people’s LTPA, which were the same as the results for the female group shown in Table 3. The results with the demographic variables included in the linear regression analysis showed that the four significant built environment elements in model 1 were still related with older people’s LTPA. Additionally, aesthetics became a new influence on older people’s LTPA, which was the same as in the female group shown in Table 4. Significant associations were also found in two demographic variables, including education level and income. Both were negatively associated with older people’s LTPA. 

## 4. Discussion

The purpose of this study was to find the association between perceived scores of built environment elements and older people’s LTPA in the city of Jinhua in the eastern region of China. Furthermore, we wanted to find the sex differences in the LTPA and-built environment association. The finding of this study could help to quantify the effect of the built environment on older people’s LTPA in a more accurate and reliable way and could provide support for decision-making in the urban construction process.

Our results showed that sex differences existed in the built environment and LTPA association. Older females’ LTPA was more sensitive to the built environment (Table 3). LTPA was affected by only two built environment elements in males, while it was affected by four elements in females. Except for land use mix diversity as a common factor in older people’s LTPA in both sex groups, all the other significant built environment factors were different. Older females’ LTPA was positively affected by residential density, street connectivity, and crime safety. However, older males’ LTPA were not affected by those built environment elements. On the other hand, access to services affected LTPA in males, but did not affect LTPA in females. The association differences that varied by gender were also found in previous studies [28,36,42,43]. Those results suggest that future studies should consider sex differences when investigating the association between older people’s LTPA and built environment elements.

The association between older people’s LTPA and the built environment was also affected by the model used. In the linear regression analysis without demographic variables, four built environment elements were related with older people’s LTPA in all participants. However, when demographic variables were included in the linear regression analysis, one more built environment element was correlated with older people’s LTPA (Table 5). This might be due to the fact that two demographic variables were found to be significantly related with older people’s LTPA, and the linear regression model was adjusted by demographic variables. Similar findings could also be observed in Table 4 in the current study and in previous studies [31,33]. Wu et al. found that the geographical location of the destination was significantly related with older people’s LTPA in Nanjing, but no significant association relationship was found after adjustment by demographic variables [31]. Indeed, older people’s LTPA can be affected by various factors. Built environment elements and demographic variables were included in this study, but psychosocial factors like self-efficacy, social support, and perceived benefits and barriers can also have an influence on older people’ LTPA [44,45,46,47]. However, it seems impossible to collect all the factors relative to older people’s LTPA in one empirical study. The association relationship without being adjusted by other factors, or with part of factors adjusted, could also explain how the older people’s LTPA were affected by the built environment to some extent. 

Land use mix diversity was a strongly influenced older people’s LTPA in this study. It was negatively associated with older people’s LTPA, and did not vary by gender and model used. The land use mix diversity element was evaluated on a 5-point Likert Scale by IPAQ-S, and a higher score means a longer distance from home to the destination. Therefore, our result indicates that a shorter distance from home to destination would motivate older people to engage more in LTPA. The average scores of land use mix diversity were 3.02 and 3.20 in the male and female group, respectively, which means that older people need 11–15 min to walk from home to destination. The results above indicate that a 11–15 min walking distance is acceptable for older people, and a shorter walking distance such as 1–5 min or 6–10 min would encourage older people to take part in more LTPA. On the other hand, walking distances such as 20–30 min and more than 30 min are not very friendly for older people. Older people might abandon their outdoor physical activity plan or select another travel mode instead of walking if the destination is not within an acceptable distance from home. A similar finding was also found in previous studies [42,48,49]. Van et al. suggested that older people’s walking/cycling behaviors were significantly positively related with perceived short distances to services in Belgium [42]. A significant difference was found in land use mix diversity between different sex groups (Table 2). Older females perceived a longer distance from home to destination than older males. 

Access to services was only positively associated with older males’ LTPA, independent of the model used. This finding is consistent with previous studies. Cerin et al. suggested that better access to shops, park/recreational facilities, public transport, and health-related destinations was positively associated with older people who walk for transport [48]. Good access to recreational facilities and parks was also found to be associated with older people’s LTPA in Barnett et al.’s study [49]. No significant association between access to services and female LTPA was found. This result might imply that access to services is not an important consideration when females take part in outdoor physical activity. The perceived score of access to services in the male group was significantly higher than that in the female group (Table 2), which suggested that older males perceived that they had better access to services. A recent report published by the World Bank suggested that because public spaces are often designed to cater primarily to men and are less accessible to women, women were 15% less likely to use public spaces and this indirectly decreased women’s physical activity [30]. Moreover, crime safety was positively associated with male LTPA after the model was adjusted with demographic variables. This positive association is consistent with previous studies [34,37,50,51]. Yu et al. found that crime safety was positively related with older people’s LTPA in the cities of Hangzhou and Wenzhou, which are also located in Zhejiang province, China [37]. However, they also supposed that because no association was found in other Chinese studies [31,36] and based on the high ranking of China in the Law-and-Order Index report published recently, the relationship between crime safety and older people LTPA in China needs more studies. 

Residential density was positively associated with older females’ LTPA, no matter what model was used (Table 3 and Table 4). No significant association was found in the older male group. The perceived score of residential density in the female group was significantly higher than that in the male group (Table 2). These results indicate that a higher residential density would motivate older females to engage more in LTPA, however, it does not affect older males. The positive association between older people’s LTPA and residential density was consistent with previous studies in China [32,37] and other countries [22,43,50,51,52,53,54]. To our knowledge, square dancing has become more and more popular in China. Residents like to take part in square dancing in crowds after dinner, and most of the participants are women. A higher residential density might help women to more easily find square dancing companions, and thus increase their LTPA. However, our results were contradicted by other Chinese studies in Xi’ an and Hangzhou [28,36]. The potential reason for the differences might be the diverse participants in these studies. Compared with adults in these two studies, older people in our study are e more able to take part in LTPA like square dancing because of more free time and less perceived pressure from work and home. Moreover, street connectivity, crime safety (Table 3), and aesthetics (Table 4) were positively related with older females’ LTPA. A convenient, safe and pleasing environment is helpful for older people to feel at ease and to overcome fear in their outdoor physical actives. Finally, it will increase older people’s LTPA and nudge them in a health-preventive direction as plenty of previous studies have suggested [51,55,56]. 

Finally, four built environment elements were found to be significantly associated with older people’s LTPA in all participants of this study, and these were also found to be significantly related with older females’ LTPA. Except for land use mix diversity, which was a common factor in both sex groups, residential density, street connectivity, and crime safety were factors only for older females LTPA. Those results mean that the associations between LTPA in older people’ and the three above built environment elements in all participants were mainly caused by the significant associations found in the female group. On the other hand, those results also indicated that LTPA in older females was more sensitive to the built environment in Jinhua. Compared with older males, older females consider more built environment elements when they take outdoor physical activity. Additionally, income situation was also found to be negatively associated with older females’ LTPA (Table 4), which suggests that older females with a lower income would engage more in LTPA. Hence, examining the association between older people’s LTPA and the built environment by gender is a better way to investigate how the built environment affects LTPA in older people. 

The strength of this study is that we investigated the association between older people’s LTPA and the built environment, especially by focusing on the association differences in different sex groups. We found that sex differences exist in the association between older people’s LTPA and the built environment. These sex differences cannot be neglected and they provide a more accurate explanation of the effect of built environment on older people’s LTPA. Secondly, most previous studies in China have targeted been at cities of super large-scale or large-scale that rank in the top 20 in terms of population size and economic development [28,31,32,33,34,35,36,37]. Few of them have focused on mid-sized cities [37]. The social-economic status of the population undoubtedly has an effect on the association between older people’s LTPA and the built environment. The current study expanded the scientific base about this association in cities of midscale. However, the present study has several limitations. Firstly, although we conducted a one-to-one interview to avoid deviations as much as possible in the data collection process, some source bias was inevitably included in the data and might influence the association relationship between older people’s LTPA and built environment; this is mainly because self-reporting tools and a cross-sectional survey of random samples of older people were used in this study. Secondly, only demographic variables were included into the linear regression analysis in this study, and social and psychological variables were not combined to explain the effect of the built environment on LTPA in older people. Those variables have been suggested to be related with older people’s LTPA in previous studies [44,46,47,56]. Thirdly, the sample size in this study was a little smaller than those in previous Chinese studies [28,31,32,33,36,37], and this might influence the association between older people’s LTPA and the built environment. Whether the associations found in Jinhua would be changed after enlarging the sample size needs further study. Additionally, the results of this paper should be extended to other populations and cultures with caution because of differences in the demographic profile and social-economic status of participants.

## 5. Conclusions

Older people’s LTPA was affected by various built environment elements in different sex groups, and older females’ LTPA level was more sensitive to the built environment. Older females consider more built environment factors when they take outdoor physical activity. Higher residential density, better street connectivity and higher crime safety would only encourage older females to take part in LTPA. A shorter perceived distance from home to destination would also motivate older people to engage in LTPA, independent of gender. Otherwise, better access to services would encourage older males to take part in LTPA. The finding of this study helps to quantify the effect of the built environment on older people’s LTPA in a more accurate and reliable way.

In order to develop an age-friendly environment in the city of Jinhua, the policy makers should take several vital built environment elements into consideration in the urban construction process as follows. Land use mix diversity is an important and common factor for encouraging older people to engage in LTPA in both sex groups. Additionally, the differences in the effect of the built environment on older people’s LTPA should also be considered by policy makers in order to increase the level of LTPA in both older males and females. A better built environment with access to service for older males and better street connectivity, crime safety and higher residential density for older females would encourage them to take part in outdoor activities. More studies are needed to investigate the association between older people’s LTPA and the built environment in mid-scale or even smaller-scale cities in China. 

## Figures and Tables

**Table 1:** Demographic characteristics of the study participants in Jinhua (*n* = 240).

Variable	Men (*n* = 132) *n*	*%*	Women (*n* = 108) *n*	*%*	*X* ^2^	*p*
Age					4.35	0.11
60–69 years	56	42.4	60	55.6		
70–79 years	60	45.5	36	33.3		
≥80 years	16	12.1	12	11.1		
Education level					21.17	<0.001 *
Primary or below	68	51.5	80	74.1		
Lower secondary	56	42.4	20	18.5		
Upper secondary	4	3	8	7.4		
Tertiary and above	4	3	0	0		
Income situation (RMB)					53.41	<0.001 *
≤1500	20	15.2	0	0		
1501–2500	4	3	36	33.3		
2501–3500	36	27.3	32	29.6		
3501–4500	40	30.3	20	18.5		
≥4501	32	24.2	20	18.5		
Travel mode choice					11.07	0.004 *
Car or bus	108	81.8	68	63		
Bicycle	16	12.1	24	22.2		
Walking	8	6.1	16	14.8		
Motion sickness					0.62	0.43
Yes	52	39.4	48	44.4		
No	80	60.6	60	55.6		

Note: * indicates significant differences between sexes in Jinhua.

**Table 2:** Comparison of older people’s leisure-time physical activity (LTPA) and perceived built environment scores for men and women in Jinhua.

Variable.	Men	Women	*t*	*p*
LTPA (MET. min/week)	2789.27 ± 1949.72	3137.22 ± 2372.39	−1.22	0.22
Residential density	598.76 ± 122.53	650.19 ± 155.11	−2.80	0.006 *
Access to services	2.86 ± 0.23	2.75 ± 0.25	3.37	0.001 *
Street connectivity	3.31 ± 0.49	3.30 ± 0.49	0.27	0.79
Walking/cycling facilities	2.97 ± 0.31	2.97 ± 0.32	−0.08	0.93
Aesthetics	2.65 ± 0.45	2.63 ± 0.37	0.33	0.74
Pedestrian/traffic safety	2.55 ± 0.56	2.44 ± 0.61	1.34	0.18
Crime safety	2.64 ± 0.54	2.73 ± 0.33	−1.63	0.11
Land use mix diversity	3.02 ± 0.52	3.20 ± 0.54	−2.77	0.006 *

Note: * indicates significant differences between sexes. MET represents metabolic equivalent score.

**Table 3:** Association between the built environment and older people’s LTPA in different sex groups.

Variable	*B*	Men*SE*	*p*	*B*	Women*SE*	*p*
Residential density	0.12	1.76	0.94	5.63	1.24	<0.001 *
Access to services	2311.60	710.15	0.001*	−1439.74	763.25	0.06
Street connectivity	237.54	393.86	0.55	1432.46	520.52	0.007 *
Walking/cycling facilities	−221.45	633.91	0.73	−1140.01	677.21	0.10
Aesthetics	−370.51	460.08	0.42	750.54	568.50	0.19
Pedestrian/traffic safety	−324.34	393.49	0.41	−149.57	298.83	0.618
Crime safety	564.90	384.11	0.14	1949.78	645.50	0.003 *
Land use mix diversity	−1234.37	359.68	0.001*	−1497.49	325.48	<0.001 *

Note: Depend variable: total score of older people LTPA, B stands for regression coefficient, SE represents stand error, * represents significant difference (*p* < 0.05).

**Table 4:** Association between the built environment and older people’s LTPA with demographic variables as a covariable in different sex groups.

Variable	*B*	Men*SE*	*p*	*B*	Women*SE*	*p*
Residential density	0.001	1.91	1.00	5.10	1.24	<0.001 *
Access to services	2553.58	727.22	0.001*	−895.73	763.25	0.29
Street connectivity	372.87	421.97	0.38	1067.90	520.52	0.06
Walking/cycling facilities	−644.11	653.17	0.33	−578.07	677.21	0.51
Aesthetics	259.99	512	0.61	1910.19	568.50	0.02 *
Pedestrian/traffic safety	−651.39	412.93	0.12	−170.46	298.83	0.60
Crime safety	791.36	394.44	0.05*	875.78	645.50	0.36
Land use mix diversity	−1373.85	406.48	0.001*	−1494.55	325.48	<0.001 *
Age	−119.45	269.12	0.66	296.48	348.09	0.40
Education level	−459.09	248.30	0.07	401.08	430.75	0.35
Income situation	−225.86	149.33	0.13	−588.94	271.65	0.03 *
Travel mode choice	−85.07	339.25	0.80	323.51	335.01	0.34
Motion sickness	619.43	374.09	0.10	−840.03	562.36	0.14

Note: Depend variable: total score of older people LTPA, B represents the regression coefficient, SE indicates the stand error, * represents significance (*p* < 0.05).

**Table 5:** Association between the built environment and older people’s LTPA in all participants.

Variable	B	Model 1SE	*p*	B	Model 2SE	*p*
Residential density	3	0.99	0.003 *	3.88	1.03	<0.001 *
Access to services	572.10	499.37	0.25	968.80	505.46	0.06
Street connectivity	612.14	311.25	0.05 *	713	311.04	0.02 *
Walking/cycling facilities	−626.70	457.34	0.17	−916.62	480.46	0.06
Aesthetics	255.75	334.39	0.45	782.04	358.34	0.03 *
Pedestrian/traffic safety	−84.32	220.12	0.10	−230.46	231.65	0.32
Crime safety	895.23	284.11	0.003 *	711.38	288.92	0.01 *
Land use mix diversity	−1115.59	234.90	<0.001*	−1304.62	241.18	<0.001*
Age				204.11	198.83	0.31
Education level				−471.29	191.62	0.02 *
Income situation				−304.78	126.11	0.02 *
Travel mode choice				−106.58	211.72	0.62
Motion sickness				222.79	278.67	0.43

Note: Model 1 only includes the built environment variables, Model 2 incorporates the built environment variables and demographic variables. Dependent variable: total score of older people LTPA, B represents the regression coefficient, SE stands for the stand error, * represents significance (*p* < 0.05).

## Data Availability

Data available on request due to restrictions privacy. The data presented in this study may be available on request from the corresponding author and with authorization of funding origination.
